# Electroencephalographic insights into the pathophysiological mechanisms of emergence delirium in children and corresponding clinical treatment strategies

**DOI:** 10.3389/fphar.2024.1349105

**Published:** 2024-06-19

**Authors:** Xin Gao, Zhichao Li, Jun Chai, Si Li, Xuanyuan Pan, Jie Liu, Linxing Li, Shangyuan Qin, Yihan Kang, Youzhuang Zhu

**Affiliations:** ^1^ Department of Anesthesiology, Shengjing Hospital of China Medical University, Shenyang, China; ^2^ Cancer Hospital, Chinese Academy of Medical Sciences, Beijing, China; ^3^ Department of Anesthesiology, Liaoning Cancer Hospital and Institute, Shenyang, China; ^4^ Department of Anesthesiology, The First Affiliated Hospital of China Medical University, Shenyang, China; ^5^ Department of Anesthesiology, The Affiliated Hospital of Qingdao University, Qingdao, China

**Keywords:** emergence delirium, neurophysiology, electroencephalography, pediatrics, general anesthesia, treatment

## Abstract

Emergence delirium is a common postoperative complication in patients undergoing general anesthesia, especially in children. In severe cases, it can cause unnecessary self-harm, affect postoperative recovery, lead to parental dissatisfaction, and increase medical costs. With the widespread use of inhalation anesthetic drugs (such as sevoflurane and desflurane), the incidence of emergence delirium in children is gradually increasing; however, its pathogenesis in children is complex and unclear. Several studies have shown that age, pain, and anesthetic drugs are strongly associated with the occurrence of emergence delirium. Alterations in central neurophysiology are essential intermediate processes in the development of emergence delirium. Compared to adults, the pediatric nervous system is not fully developed; therefore, the pediatric electroencephalogram may vary slightly by age. Moreover, pain and anesthetic drugs can cause changes in the excitability of the central nervous system, resulting in electroencephalographic changes. In this paper, we review the pathogenesis of and prevention strategies for emergence delirium in children from the perspective of brain electrophysiology—especially for commonly used pharmacological treatments—to provide the basis for understanding the development of emergence delirium as well as its prevention and treatment, and to suggest future research direction.

## 1 Introduction

Emergence delirium (ED) in children is defined as the impairment of a child’s attention and awareness of the environment with disorientation and altered perception, including hypersensitivity to stimuli and hyperactive motor behavior, which occurs immediately after surgery. Children with ED exhibit irritability, uncooperativeness, sadness, crying, moaning, writhing, and kicking (Eckenhoff et al., 1961). Although most ED cases are self-limiting or present as a benign process (Jalili et al., 2019), it may cause unnecessary violence and self-harm, prolong the stay in the post-anesthesia care unit (PACU) affect the child’s postoperative recovery, cause parental dissatisfaction, and increase medical costs (Urits et al., 2020; Simonini et al., 2021). Additionally, children, 1 week after surgery, are more likely to experience behavioral changes such as maladjustment, insomnia, bedwetting, and lack of attention (Urits et al., 2020). One pilot study found no significant long-term effects of pediatric delirium on overall cognition, executive function, or behavior; however, baseline levels of cognition and behavior were not assessed (Meyburg et al., 2018). More prospective trials are needed to confirm and analyze the recovery process of brain function and consciousness after pediatric delirium, and its long-term adverse effects. Therefore, research on the mechanisms and prevention of ED is crucial.

Several studies have explored the mechanisms of ED, finding that it is associated with inhaled anesthetics (such as sevoflurane), preschool age, type of surgery (including eye, ear, nose, and throat surgery), pain, preoperative anxiety, psychological immaturity, personality/temperament, and physiological conditions (Moore and Anghelescu, 2017) (Table 1). The incidence of ED in children after sevoflurane anesthesia ranges from 10% to 80%, which is 3–8 times higher than that in adults (Urits et al., 2020). Among them, in preschool and school-age children, the incidence is 40% and 11.5%, respectively (Dahmani et al., 2014). Currently, the most prevailing hypothesis for the occurrence of ED in patients under general anesthesia is that the clearance rate for these agents varies between different sites in the central nervous system, resulting in different rates of functional recovery at these sites. Compared to other brain functions (auditory and motor), cognitive function recovers late, and variations in brain function recovery contribute to the occurrence of ED (Dahmani et al., 2014).

**TABLE 1:** Risk factors for ED and diagnostic and treatment strategies.

ED scale	Contributors to ED	Strategies to decrease ED
• PAED	• Volatile anesthetics	• Propofol
• Watcha	• Type of surgery	• Dexmedetomidine
• CAM-ICU	• Age	• Ketamine
• EAS	• Pain	• Magnesium
• DSM	• Patient anxiety	• Opioids
	• Patient characteristics	• Acupuncture
	• Pre-existing behavior	• Others (benzodiazepines/clonidine/gabapentin/humanistic care/regional anesthesia)
	• Physiological status	

ED, emergence delirium; PAED, pediatric anesthesia emergence delirium; Delirium Assessment Scale for the Intensive Care Unit; EAS, emergence agitation score; DSM, diagnostic and statistical manual of mental disorders.

Brain function is signalized in the form of brain electricity, the generation and conduction of neuronal action potentials and inter-synaptic transmission are the basis of neurophysiological activity, and brain waves are the overall reflection of the electrophysiological activity of the central nerve cells in the cerebral cortex or on the surface of the scalp. Changes in brain electricity are the basis of brain function. Many drugs can reduce the incidence of ED, such as dexmedetomidine, fentanyl, ketamine, and propofol supplementation at the end of sevoflurane anesthesia (Ng et al., 2019; Jiao et al., 2020; Haile et al., 2021; Zhang et al., 2022)). The most recognized effective adjuvant drug is dexmedetomidine, which has intrinsic sedative and analgesic effects. These drugs cause patient-specific electroencephalographic (EEG) changes to achieve ED prevention and control.

**FIGURE 1:**
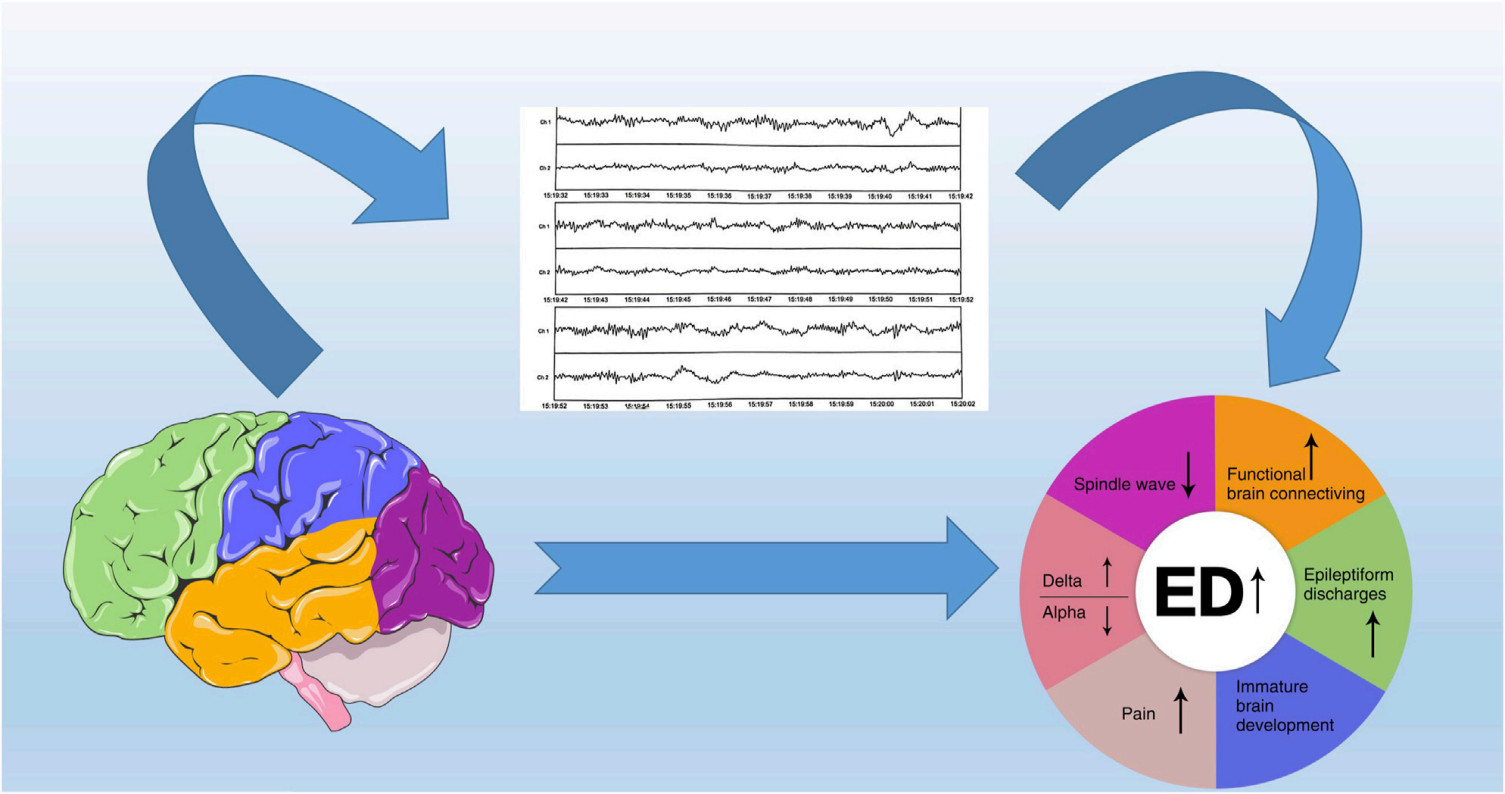
The electroencephalogram of emergence delirium mechanism.

EEG is a direct and real-time measurement of neural activity in the brain, responding to brain changes in real time. There is a lack of evidence on the use of EEG to predict ED in children. In this paper, we will provide an overview of the mechanisms and preventive measures of ED from the perspective of changes in brain wave power during awakening from anesthesia, alterations in functional connectivity of the brain, abnormal discharges during the induction period of anesthesia, physiological development of the brain, and pain (Figure 1). The adverse effects of general anesthesia on children are manifested by a series of different behavioral abnormalities experienced during the awakening period, including ED, emergence excitation, and emergence agitation. These terms are not synonymous but are used interchangeably to describe behavioral disorders following anesthesia, especially in pediatric patients, because it is difficult to clearly define the mental state and behavior of children during the awakening period (Mason, 2017; Larsen et al., 2022). Herein, we uniformly use ED to describe abnormal behaviors during the waking period.

## 2 Physiological EEG and anesthesia EEG

### 2.1 EEG in the physiological state

The basic EEG waveforms are alpha, beta, theta, gamma, and delta waves. Some normal EEG waves with special waveforms—such as hump, σ wave, λ wave, k-complex wave, and μ wave—can also appear during sleep. During sleep, the EEG changes are divided into two states: non-rapid eye movement (NREM) and rapid eye movement (REM) sleep. The first stage of NREM sleep is dominated by theta and beta waves, which is the transition stage between full wakefulness and sleep. Spindle and k-complex waves dominate the second stage of NREM sleep. The spindle wave is a typical marker of sleep, appearing when consciousness disappears. It does not appear during wakefulness or REM sleep and is abnormally reduced in insomnia. Notably, the sleep spindle wave, one of many important measurable indicators of brain activity during sleep, provides a window into the current state of brain health and an individual’s risk for brain disease or cognitive decline. Slower delta waves begin to dominate during stage III NREM sleep; stage IV is entered when delta waves comprise more than 50% of the EEG waves. The EEG reveals a mixture of low frequency and low amplitude activity, including beta, theta, and alpha waves (Brown et al., 2012; Schwartz and Kilduff, 2015) (Table 2). In conclusion, the waveforms of brain waves are diversified and dynamically change with the changes of brain consciousness and sleep cycles, and the EEG waveforms are consistent with the conscious state of the brain, which is an important indicator for monitoring the central conscious activity and arousal state.

**TABLE 2:** EEG classification based on wave frequency and sleep characteristics.

Waveform	Features	Occurrence status	Anomalies
Alpha wave	Frequency range: 8–13 Hz Amplitude: 20–100 µV	Awake state, but with closed eyes to relax	If present in the frontal region, depression and attention problems may be suspected; head injury
Beta wave	Frequency range: 13–30 Hz Amplitude: 5–20 µV	State of alertness, or engaging in mental activity; when taking medication	Absence may lead to poor concentration and problem-solving skills; high activity can lead to sleep disturbance, hyperactivity
Theta wave	Frequency range: 4–8 Hz Amplitude greater than beta wave	Sleepy state; early stages of sleep	Suspected head injury and brain lesions if present in awake adults
Delta wave	Frequency range: 0.5–4 Hz Amplitude: 20–200 µV	Deep dreamless sleep, non-rapid eye movement sleep; subconscious	Possible brain lesion or tumor if present in a conscious adult; possible injury secondary to stroke
Gamma wave	Frequency range: ≥30 Hz Small amplitude	Physical movement; higher level of mental activity	When working continuously for a long time
k-complex wave	Delta frequency, large amplitude, spikes, symmetrical; followed by rhythmic theta waves; occurs in the bifrontal region	Each time one is partially awakened from sleep; the second stage of sleep	If it is detected while awake, it is abnormal
λ wave	Bilateral positive waves, triangular in shape, generally symmetrical; occur in the occipital region	When one is awakened; staring at a blank, uniform interface, such as when reading and watching TV	Abnormal if found during concentration or problem-solving
Spindle wave	Higher frequency than alpha waves or lower frequency than beta waves, with a duration of seconds or less; amplitude first increases and subsequently slowly decreases with symmetry; found in the parasagittal region	During the second stage of sleep	If fusiform waves are detected in the awake state, it is considered a seizure
μ wave	Asymmetrical, rhythmic, rounded in one direction, sharp in the other, half the frequency of fast beta wave activity; seen in the parasagittal region	When the cerebral cortex is exposed or the individual has structural brain defects	Abnormal if this wave is present after a motor action is completed

EEG, electroencephalogram.

### 2.2 EEG under anesthesia

To elucidate the relationship between anesthesia and EEG, Chander et al. used standardized terms to define specific EEG changes during anesthesia maintenance, similar to those used to describe natural sleep: slow-wave anesthesia (SWA), predominantly delta and spindle wave activity, non–slow-wave anesthesia (NSWA), very low spindle wave and delta power, delta-dominant slow-wave anesthesia (ddSWA), and spindle-dominant slow-wave anesthesia (sdSWA)) (Chander et al., 2014). The spectral power corresponding to the four fundamental EEG frequency ranges was integrated to obtain the absolute power of each wave. The relative power (RP) of each wave is the ratio of the absolute power of each wave to the sum of the powers in all EEG frequency ranges. From the beginning of the awakening period (cessation of drug administration) to the end, approximately half of the patients showed typical EEG phase changes: the absence of delta waves followed by the absence of spindle waves followed by a period of NSWA (Chander et al., 2014). The change in consciousness level from anesthesia sleep to wakefulness is customarily accompanied by a gradual transition between low- and high-frequency EEG oscillations at multiple frequencies and closely resembles awakening from natural sleep.

Sevoflurane is a commonly used pediatric anesthetic, and like propofol, enhanced GABAergic inhibition may be its primary mechanism of action. At sub-MAC concentrations, sevoflurane exhibited EEG manifestations similar to those of propofol, enhanced GABAergic inhibition of the thalamic reticular nucleus (TRN)to reduce excitatory inputs from the thalamus to the cortex, resulting in alpha oscillations and beta oscillations; enhanced inhibition of GABAergic projections to brainstem arousal centers, and enhanced hyperpolarization of cortical pyramidal neurons, which facilitated the emergence of slow oscillations and delta oscillations. In addition, sevoflurane also blocks K+ channels and hyperpolarization-activated cyclic nucleotide-gated cation channels, binds to NMDA receptors, and blocks the release of glutamate (Cohen et al., 2003; Hemmings et al., 2005; Moore and Anghelescu, 2017). When the concentration of sevoflurane is increased to the level of MAC and above, theta oscillations will also occur, forming a unique pattern of uniform distribution from the slow oscillation range to the alpha range (Purdon et al., 2015).

## 3 ED and EEG

### 3.1 ED and delta and alpha waves

Recently, several studies have suggested that the occurrence of postoperative delirium (POD) in the elderly is closely related to high delta RP and low alpha RP (van der Kooi et al., 2015; Gutierrez et al., 2019; Numan et al., 2019; Lutz et al., 2022). Lutz et al. (Lutz et al., 2022) looked at the EEG performance of the frontal lobe during the awakening period under static-absorbent combined anesthesia in patients (age >18 years) undergoing elective surgery, and found that patients who developed delirium in the PACU had higher delta RP and lower alpha RP.

Due to incomplete brain development, the EEG characteristics of children differ greatly from those of adults (Cornelissen et al., 2018). More clinical and preclinical evidence is needed to clarify whether the adult findings can be directly extrapolated to children. Bruni et al. found that children exhibited crying, screaming, and night terrors when continuous delta activity was interrupted during NREM sleep. This is similar to the manifestations of irritability, uncooperativeness, sadness, crying, moaning, writhing, and kicking in children with delirium. We speculate that the occurrence of ED in children may be related to continuous delta frequency activity.

Kim et al. observed the two-channel frontal EEG of 60 children aged 2–10 years undergoing ophthalmological or otorhinolaryngological surgery under sevoflurane maintenance anesthesia. Although the emergence trajectories did not differ significantly between the ED and non-ED groups, the frontal delta RP during emergence was greater, and the alpha and beta RPs were lower, in the ED group. This is similar to the EEG trajectory during emergence found in adults with ED as mentioned earlier. Additionally, they found that the ratio of low- (delta and theta) to high-frequency (alpha and beta) waves, the delta/alpha ratio, was positively correlated with PAED scores (Kim et al., 2021). The results of this study further confirm that the occurrence of ED in children somewhat resembles that in adults, and that the increased delta/alpha ratio during emergence is an important factor in the occurrence of ED in pediatric patients.

However, Jang et al. (Jang et al., 2018) concluded that children with a decreased theta/alpha ratio during the awakening period had a high risk of postoperative ED, which may be caused by the small sample size of his study, different assessments of ED (Sikich and Lerman, 2004), and different definitions of the awakening period. We believe that the high delta/alpha ratio during emergence is suggestive of ED occurrence.

### 3.2 ED and spindle waves

The spindle wave is a characteristic sleep EEG wave of NREM stage II and occurs after loss of consciousness. The mechanism of spindle wave generation based on the recruitment of T-type calcium channels in the thalamocortex (TC) and TRN has been widely accepted. Activation of T-type calcium channels on GABAergic neurons in the TRN generates a low-threshold calcium potential and the release of GABA from the GABAergic neurons acts on the glutamatergic neurons in the TC to generate a GABAAipsp, which is followed by the Restoration of the membrane potential triggers the opening of T-type calcium channels on glutamatergic neurons, releasing glutamatergic neurotransmitters, and glutamate in turn causes neurons in the NRT to produce EPSP, which helps trigger electrical activity at the spindle frequency (Crunelli et al., 2014). In addition, Astori found that CaV3.3 channels have a central role in generating sleep spindle wave rhythms (Astori et al., 2011). By performing frontal EEG in 626 patients undergoing general anesthesia for elective non-cardiac surgery, Hesse et al. found that those with no sdSWA emergence trajectories had an increased probability of ED in the PACU, whereas those with sdSWA emergence trajectories had a decreased probability (Hesse et al., 2019). This suggests that the occurrence of ED is not only associated with an increase in delta RP and a decrease in alpha RP but is also inextricably linked to a decrease in spindle wave power. We hypothesize that the decrease in spindle wave power during emergence from general anesthesia also contributes to the occurrence of ED. However, there is a lack of studies on the correlation between spindle wave changes during pediatric emergence from general anesthesia and the incidence of ED after surgery, which is one of our future research directions.

### 3.3 ED and functional brain connectivity

The human brain has a high density of short connections within brain regions and sparse long connections between brain regions. This property allows the real-time transmission of messages between multiple systems, an efficient organization of internal and external information, and an efficient exchange of information between different functional brain regions, i.e., the network property reflects the information exchange property of brain functional differentiation and integration. There are four types of “connectivity” frequently measured in the brain (Mashour and Avidan, 2014). In 1993, Friston et al. defined functional connectivity as the temporal correlations between spatially remote neurophysiological events (Friston et al., 1993). Functional brain connectivity is a measure of temporal correlations and functional activity dependencies between spatially separated brain regions and is one of the effective means of describing synergistic working patterns between brain regions. Additionally, there exists structural, directional, and efficiency connectivity (Mashour and Avidan, 2014). What is the relationship between the occurrence of ED during anesthesia awakening and the functional connectivity of the brain?

In 2012, Choi et al. used a functional magnetic resonance imaging (MRI) seed-based correlation and found increased functional connectivity between the prefrontal cortex and posterior cingulate cortex during the onset of delirium in 22 patients (Choi et al., 2012). In 2014, Martin et al. used multichannel EEG to monitor the EEG activity of children during the emergence period and used global efficiency (GE) and global coherence of the whole brain, frontal lobe, and parietal lobe as the response form of brain functional connectivity. The whole brain and parietal functional connectivity did not significantly differ between the ED and control group, although the mean GE values for the frontal network were significantly higher in the ED group. This study suggests that increased connectivity after discontinuation of sevoflurane anesthesia may be closely related to the development of ED (Martin et al., 2014). However, given the small sample size of this study, individual differences, condition restrictions, and preoperative medications may have significantly affected the experimental results. Thus, more rigorous studies with larger sample sizes are needed to further explore the relationship between functional brain connectivity and ED during anesthesia awakening and the related molecular mechanisms in the future. Additionally, Dellen et al. analyzed the functional connectivity of EEG time series using the phase lag index, directed phase lag index, and functional brain network topology with graph analysis, and found that patients with delirium after cardiac surgery had a loss of functional connectivity in the alpha band, reduced pathway length, and increased connectivity in the delta band (van Dellen et al., 2014). This is consistent with our previous findings regarding the relationship between alpha and delta waves and ED, suggesting a potential association between ED and altered functional connectivity of the brain, in addition to specific cortical sites, and specific EEG frequency fractions.

Therefore, increased functional connectivity may reflect a state of cortical hyperexcitability, and greater network connectivity implies increased responsiveness to internal and external stimuli. This increased responsiveness may be an important cause of ED.

### 3.4 ED and epileptiform discharges

Recent studies have found that epileptiform discharges during general anesthesia induction are also involved in the mechanism of ED. Epileptiform discharges are abnormal waveforms on the EEG that indicate the possible presence of epilepsy and manifest as sharp waves, spike waves, sharp-and-slow-wave complexes, or spike-and-slow-wave complexes. A sharp wave is a triangle wave with an 80–300 ms time limit; a wave amplitude of 200 µV or more; a fast, straight rise; and a slow fall. A spike wave is an abnormal waveform with a 20–70 ms time limit, an amplitude exceeding 20 μV, a steep rise, and falling branches, and an overall thorn shape. Sharp and spike waves can be combined with slow waves. Some studies have reported that epileptiform discharges are frequently observed during general anesthesia induction with sevoflurane (Vakkuri et al., 2001; Schultz et al., 2012). According to Vakkuri et al., the following types of epileptiform discharges were described during sevoflurane induction: delta with spikes (DSP), rhythmic polyspikes (PSR), periodic epileptiform discharges (PED), and suppression with spikes (Vakkuri et al., 2001). Moreover, Kreuzer et al. found a reduction in EEG epileptiform activity in children anesthetized with 6% sevoflurane compared with 8% sevoflurane (Kreuzer et al., 2014). Hesdorffer et al. found that most children with attention deficit disorder and hyperactivity develop epileptiform activity on EEG (Hesdorffer et al., 2004). This suggests that epileptiform activity may be associated with behavioral hyperactivity. ED is characterized by hypersensitivity to stimuli and hyperactive motor behavior, leading us to hypothesize that epileptiform EEG is most likely associated with ED.

Koch et al. analyzed dual-channel EEG signals during anesthesia induction in 62 children aged 0.5–8 years and found that epileptiform discharges were more common in children with ED. They concluded that the occurrence of ED in children was significantly associated with epileptiform discharges during general anesthesia induction, especially PSR, PED, and DSP. They also confirmed a higher incidence of epileptiform discharges during sevoflurane than propofol anesthesia-induced loss of consciousness (Koch et al., 2018). Interestingly, the incidence of epileptiform discharges induced by propofol and sevoflurane was consistent with the clinical occurrence of ED, indirectly confirming the correlation between epileptiform discharges during induction and ED in pediatric patients. There is insufficient evidence on the correlation between EEG performance during induction and the occurrence of ED when comparing general intravenous anesthesia with sevoflurane inhalation anesthesia—this is a direction for our future research.

### 3.5 ED and physiological brain development

Voepel-Lewis et al. assessed 521 children who underwent outpatient surgery and found that age was an independent risk factor for ED—the younger the child, the higher the risk (Voepel-Lewis et al., 2003). However, during infancy and early childhood, EEG performance under sevoflurane anesthesia differs from that of older children and adults because the nervous system is rapidly developing. Cornelissen et al. found slow oscillations (0.1–1 Hz) and delta oscillations (1–4 Hz) on the EEG of neonates, infants, and toddlers during sevoflurane general anesthesia, and the consistency of slow and delta oscillations was the highest at 3 months; thereafter, it decreased and became discordant at 9 months. Alpha oscillations appear in the frontal lobe at approximately 3–4 months and increase steadily until they reach a plateau and become coherent at 10 months. That is, infants have coordinated local frontal slow and delta oscillations during the first 8 months of life, while from 10 months, fine-tuning of coordinated alpha oscillations begins to appear, resembling an adult EEG (Cornelissen et al., 2018). Therefore, the EEG mechanisms underlying the occurrence of ED in infants and children need to be analyzed considering the EEG characteristics caused by age itself, which may be one of the mechanisms behind the large differences in ED incidence between adults and children.

### 3.6 Pain and EEG

Recently, several studies have shown that pain is an independent risk factor for the development of ED (Moore and Anghelescu, 2017; Wei et al., 2021). Gross et al. found that painful stimuli induced gamma oscillations in the primary somatosensory cortex by laser stimulation of the right dorsum in healthy volunteers, and that the amplitude of these oscillations increased with increasing objective stimulus intensity and subjective pain intensity (Gross et al., 2007). Similarly, Baroni et al. observed increased gamma activity in the central and frontal regions of adult patients with chronic orofacial pain compared with controls (Baroni et al., 2020), while Buchanan et al. found significantly higher delta and theta power in patients with post-concussive syndrome and chronic pain following a motor vehicle collision compared with controls (Buchanan et al., 2021). Hence, pain may cause changes in various EEG activities. Zis et al. reviewed 20 relevant articles and found that EEG performance was consistent with an increase in delta and gamma wave power, despite inconsistent conclusions on the relationship between delta, theta, alpha, beta, and gamma activities and painful stimuli due to differences in individuals, stimulus intensity, stimulus location, and state at the time of recording (eyes open/closed). The authors concluded that these increases are characteristic EEG manifestations of nociception (Zis et al., 2022). This led us to further confirm that pain causes changes in brain waves, with enhanced changes in delta and gamma wave activity in particular. We hypothesize that once anesthesia is discontinued after surgery, pain caused by surgical stimulation and intolerance caused by artificial ventilation devices (e.g., tracheal tubes) would cause EEG changes during the emergence period. This could lead to an increase in delta and gamma wave power, which could lead to the development of ED.

Hence, EEG delta and alpha wave power imbalance during emergence from general anesthesia, decreased or absent spindle waves, increased functional brain connectivity, epileptiform discharges during anesthesia induction, brain underdevelopment, and pain-induced increases in EEG delta and gamma wave power are all involved in the pathogenesis of pediatric ED. The electrophysiological mechanism of pediatric ED in the brain is based on multiple factors. A single factor cannot clarify the pathogenesis of ED (Table 3)—various aspects have not yet been investigated, and extensive basic and clinical neurophysiological studies are still required.

**TABLE 3:** EEG-based mechanisms of ED onset.

Author	Year	Population	N	Trial	Conclusions
van der Kooi et al. (2015)	2015	Patients aged ≥50 years who were to undergo cardiothoracic surgery	56	Single-center observational study	In a homogenous population of non-sedated patients, it was observed that relative delta power from an eyes-closed EEG obtained with only two electrodes in a frontal–parietal derivation can distinguish among patients who have delirium and those who do not
Numan et al. (2019)	2019	Patients aged ≥60 years, planned major surgery with an expected stay of at least 2 days	159	Prospective multicenter cohort study	Single-channel EEG can detect delirium/possible delirium in older post-operative patients and is a promising method for the routine detection of delirium
Gutierrez et al. (2019)	2019	Patients aged >60 years who were scheduled for elective major abdominal surgery	36	Exploratory observational study	A low intraoperative alpha power is a novel EEG marker to identify patients who will develop alterations in CAM— i.e., with PD or PSSD—after surgery
Lutz et al. (2022)	2022	Patients aged >18 years who underwent elective surgery under general anesthesia	201	Prospective observational study	Differences in EEG features associated with anesthesia emergence in patients with and without PACU-D were reported. Frontal and global EEG alpha-band features could help to identify patients with PACU-D
Jang et al. (2018)	2018	Children aged 1–6 years scheduled for strabismus surgery	31	Prospective cohort study	Children showing high alpha power and low theta power (low theta/alpha ratio) during emergence from sevoflurane anesthesia are at high risk of EA in the PACU.
Kim et al. (2021)	2021	Children aged 2–10 years undergoing elective ophthalmological or otorhinolaryngological surgery requiring general anesthesia	60	Prospective, single-center, cohort study	Pediatric patients developing ED have increased low-frequency (δ) frontal EEG activity with reduced high-frequency (α and β) activity during emergence from general anesthesia
Hesse et al. (2019)	2019	Patients admitted to the PACU after receiving general anesthesia for non-emergency, non-cardiac surgery	626	Multicenter observational study	EEG emergence trajectories lacking significant spindle power were strongly associated with PACU-D
Choi et al. (2012)	2012	Patients with delirium who were able to cooperate with MRI scanning procedures	22	Observational study	The disruption in the reciprocity of the dorsolateral prefrontal cortex with the posterior cingulate cortex and reversible reduction of functional connectivity of subcortical regions may underlie the pathophysiology of delirium
Martin et al. (2014)	2014	Children aged 5–15 years undergoing minor elective surgery procedures	12	Cohort study	ED is associated with arousal from an indeterminate state before the onset of sleep-like EEG patterns
van Dellen et al. (2014)	2014	Patients aged ≥50 years undergoing cardiac surgery	54	Cross-sectional, observational, single-center study	Loss of α band functional connectivity, decreased path length, and increased δ band connectivity directed to frontal regions characterize the EEG during delirium after cardiac surgery
Koch et al. (2018)	2018	Children aged 0.5–8 years, undergoing elective surgery with a planned duration of at least 60 min	412	Prospective, observational cohort study	ED in children is significantly related to interictal spike events occurring during induction of anesthesia
Cornelissen et al. (2018)	2018	Children aged 0–3 years requiring elective surgery below the neck	91	Observational study	Key developmental milestones in the maturation of the thalamocortical circuitry likely generate changes in EEG patterns in infants undergoing sevoflurane general anesthesia. Characterization of anesthesia-induced EEG oscillations in children demonstrates the importance of developing age-dependent strategies to monitor adequately the brain states of children administered general anesthetics
Gross et al. (2007)	2007	Healthy men	12	Observational study	Painful stimuli induce gamma oscillations in the contralateral S1 cortex
Baroni et al. (2020)	2020	Patients with temporomandibular disease	48	Cross-sectional study	Abnormal EEG activity was recorded during painful stimulation compared with the relaxed condition in patients with chronic orofacial pain as well as in healthy controls
Buchanan et al. (2021)	2021	Patients with physician-diagnosed chronic pain and post-concussion syndrome with onset beginning after an MVA. Patients must have sustained mild traumatic brain injury due to MVA. Nearly age- and sex-matched controls	111	Matched case–control study	Distributed increases in slow-wave oscillatory power are concurrent with post-concussion syndrome and chronic pain
Zis et al. (2022)	2022	A systematic literature search in PubMed on 14 January 2021, using the following MeSH terms: Term A: “EEG” OR “electroencephalography”; Term B: “pain” OR “painful”	20	Systematic review	Although no robust EEG biomarkers of pain perception have been identified, EEG has potential, and future research should be undertaken

EEG, electroencephalography; ED, emergence delirium; PACU, post-anesthesia care unit; MRI, magnetic resonance imaging; MVA, motor vehicle accident; MeSH, medical subject headings; PD, postoperative delirium; PSSD, postoperative subsyndromal delirium; PACU-D, delirium in the post-anesthesia care unit; EA, emergence agitation.

## 4 Prevention and treatment

Currently, there are no definitive clinical methods to effectively prevent or treat ED given its unknown pathogenesis. Nonetheless, many recent clinical and preclinical studies have shown interest in the prevention and treatment of ED, and an increasing number of measures have been suggested. These include pharmacologic and nonpharmacologic treatments (Cui et al., 2020). Below, we discuss several ED prevention measures commonly used in clinical practice based on the electrophysiologic aspects of the brain mentioned above (Table 4).

**TABLE 4:** Prevention and treatment of ED.

Author	Year	Population	N	Measures	Conclusions
Ramlan et al. (2020)	2020	Children aged 1–5 years with an ASA physical status I or II undergoing surgical procedures under general anesthesia using sevoflurane	108	Propofol 0.5 mg/kg was administered intravenously at the end of anesthesia	Administration of propofol 0.5 mg/kg at the end of anesthesia effectively reduces the incidence of ED in children undergoing general inhalational anesthesia with sevoflurane
Yao et al. (2015)	2015	Children aged 3–7 years with an ASA physical status I who underwent elective unilateral strabismus surgery	90	Intranasal saline or dexmedetomidine administered approximately 45 min before sevoflurane induction	Intranasal dexmedetomidine premedication produces a dose-dependent decrease in the MACLMA of sevoflurane and the emergence delirium in the PACU.
Heinmiller et al. (2013)	2013	Children aged 6 months to 14 years undergoing strabismus surgery	50	Clonidine 2 μg/kg or a similar volume of saline was administered intravenously, preoperatively after induction	Children receiving clonidine before undergoing strabismus surgery have a small but noticeable reduction in postoperative agitation
Schmitz et al. (2018)	2018	Children aged 3 months to 10 years scheduled as outpatients for elective MRI with deep sedation	351	Ketamine 1 mg/kg was administered at induction	There were no significant differences in ED occurrence
Ali et al. (2020)	2020	Children aged 3–15 years undergoing adenotonsillectomy	120	Ketamine 0.15 mg/kg followed by propofol 0.45 mg/kg as intravenous ketofol (1:3) was administered approximately 10 min before the end of surgery	Ketofol (1:3) shows a significant ability to reduce postoperative agitation in children undergoing adenotonsillectomy
Lee et al. (2020)	2020	Children aged 2–5 years who underwent strabismus surgery	66	After anesthesia induction, the magnesium group received an initial loading dose of 30 mg/kg magnesium sulphate over 10 min and, subsequently, continuous infusion of 10 mg/kg/h until 10 min before the end of the surgery	Magnesium supplementation during anesthesia had no significant effects on the incidence of ED or postoperative pain in children undergoing strabismus surgery
Abdulatif et al. (2013)	2013	Children aged 4–7 years with an ASA physical status I or II who were scheduled for adenotonsillectomy	70	An initial intravenous loading dose of 0.3 mL/kg (30 mg/kg) of a 10% solution over 10 min was administered. This was followed by continuous infusion of 0.1 mL/kg/h (10 mL/kg/h) for the entire duration of surgery	Magnesium sulphate reduces the incidence and severity of emergence agitation in children undergoing adenotonsillectomy using sevoflurane anesthesia
Choi et al. (2018)	2018	Children aged 3–7 years with an ASA physical status I who were scheduled to undergo strabismus surgery in both eyes	80	Remifentanil was intravenously infused at a rate of 0.05 μg/kg/min until the discharge criteria were met in the PACU	Maintaining a low dose of remifentanil throughout the recovery phase attenuated the incidence of ED in children undergoing strabismus surgery under sevoflurane anesthesia
Aykut and Işık (2018)	2018	Children aged 3–8 years undergoing tonsillectomy and/or adenoidectomy	64	Midazolam 0.5 mg/kg was administered orally	Midazolam premedication reduced the frequency of ED.
Singla et al. (2021)	2021	Children aged 3–8 years who received sevoflurane anesthesia for elective ambulatory procedures	132	Children were randomized to receive oral premedication with either melatonin 0.3 mg/kg, midazolam 0.3 mg/kg, or honey as a placebo	A multimodal anxiolytic approach including oral melatonin, as opposed to oral midazolam, significantly reduced ED after sevoflurane anesthesia
Pinto Filho et al. (2019)	2019	Children aged 1–6 years who underwent myelogram or lumbar puncture	135	Two groups: 15 mg/kg vs. 30 mg/kg oral gabapentin	Gabapentin reduces the occurrence of ED and postoperative vomiting up to 8 h after the procedure
Lin et al. (2009)	2009	Children aged 1–6 years who were scheduled for BMT insertion surgery	60	Acupuncture was applied at points LI-4 (he gu) and HT-7 (shen men) immediately after induction of anesthesia	Acupuncture therapy may be effective in diminishing both pain and emergence agitation in children after BMT insertion without adverse effects
Wang et al. (2021)	2021	Children aged 3–7 years who required elective pediatric or otolaryngological surgery undergoing general anesthesia with endotracheal intubation	102	A recorded maternal voice file vs. a recorded stranger’s voice file	The recorded maternal voice could effectively alleviate the degree of ED in the recovery period and reduce the occurrence of ED.

ED, emergence delirium; ASA, american society of anesthesiologists; MRI, magnetic resonance imaging; BMT, bilateral myringotomy and tympanostomy tube; PACU, post-anesthesia care unit.

### 4.1 Decrease in delta/alpha ratio

#### 4.1.1 Propofol

In recent years, studies have found that general anesthetics are closely associated with the development of ED. Propofol and sevoflurane, the most widely used anesthetics in clinical practice, are widely used in the induction and maintenance of general anesthesia by activating the functional Cl-channels formed by the β-subunit of GABA A receptor, and the increase in the inward Cl-current causes the post-synaptic neuron to hyperpolarize and agonize the GABA A receptor, exerting central inhibitory effects (Sanna et al., 1995).

Clinical studies have shown a higher ED incidence in children induced with inhalational than with intravenous anesthetics (Peker and Polat, 2020). Furthermore, the incidence of ED after sevoflurane administration is higher than that after desflurane (Wu et al., 2019b); however, propofol can reduce the incidence of ED after sevoflurane anesthesia (Wu et al., 2019a; Ramlan et al., 2020). Wu et al. retrospectively analyzed the data of 200 children undergoing inguinal hernia, where the experimental group received 2 mg/kg propofol intravenously, 5 min after discontinuation of sevoflurane, and the control group received sodium chloride 0.9%. Using the PAED scale, they concluded that propofol administered after discontinuation of sevoflurane inhalation anesthesia could prevent the development of pediatric ED (Wu et al., 2019a). This corresponds with the results of a double-blind RCT conducted by Ramlan and colleagues. The Aono and PAED scales were used to assess the incidence and severity of ED in children aged 1–5 years in the PACU within 30 min. They found that propofol 0.5 mg/kg administration immediately after resumption of spontaneous breathing following cessation of inhaled sevoflurane was effective in reducing the incidence of ED (Ramlan et al., 2020).

Previously, Ching et al. demonstrated that propofol can stimulate increased alpha wave power in the frontal cortex as well as hypersynchronous neurobiological manifestations. They hypothesized that propofol-induced increases in thalamocortical hypersynchrony and alpha wave power may interfere with the flexible cortical communication required for brain consciousness, causing selective inhibition of frontoparietal feedback connectivity and ultimately loss of consciousness (Ching et al., 2010; Dehaene and Changeux, 2011). Therefore, combined with previous findings on the relationship between alpha waves and neurological connectivity of the brain regions and ED occurrence, we believe that the propofol-induced increased alpha wave power and reduced brain connectivity may have a protective effect on ED occurrence.

#### 4.1.2 Magnesium

Magnesium is a noncompetitive blocker of NMDA receptors (Mayer et al., 1984), reduces the incidence of postoperative shivering, and has good analgesic properties (Lysakowski et al., 2007). Clinically, the most commonly used magnesium-containing drug is magnesium sulfate (MgSO4). Recently, MgSO4 has been used as an adjunct to general anesthetic agents for sedation and analgesia, and may potentially prevent ED.

There is no consensus on whether MgSO4 can reduce the incidence of postoperative ED in children (Abdulatif et al., 2013; Lee et al., 2020; Koo et al., 2022; Shen et al., 2022). An RCT by Lee et al. included 66 children aged 2–5 years undergoing daytime strabismus surgery who received MgSO4 30 mg/kg as an initial loading dose after anesthesia induction, followed by continuous infusion of 10 mg/kg/h. They found no significant effect of magnesium supplementation during anesthesia on the incidence of ED (Lee et al., 2020). A meta-analysis by Shen et al. including 635 patients aged <18 years from eight RCTs also concluded that MgSO4 did not reduce the incidence of ED in children (Shen et al., 2022). However, Abdulatif et al. observed 65 children undergoing adenotonsillectomy with sevoflurane induction followed by intravenous administration of MgSO4 and found the incidence of ED in the experimental group and the control group was 36% and 72%, respectively. They concluded that MgSO4 reduced the incidence and severity of ED in these children (Abdulatif et al., 2013). A meta-analysis by Koo et al. on the effect of intraoperative MgSO4 administration on postoperative agitation and delirium in pediatric patients also reported that MgSO4 has a positive effect on reducing ED incidence (Koo et al., 2022). The discrepancy in the results of these RCTs may be owing to the different types of surgery—the high ED incidence following ENT surgery is more likely to deliver positive results—and the different induction methods and doses of MgSO4 administered. Overall, we believe that MgSO4 has a role in preventing the occurrence of ED in children.

Magnesium is involved in energy metabolism, preserving brain glucose or pyruvate in tissues to meet energy demands during cerebral ischemia and reperfusion, and suppressing brain lactate and elevated glutamate levels (Lin et al., 2002; Bariskaner et al., 2003). Brain tissue energy metabolism is closely related to EEG grading. An increase in EEG grading is manifested by a decrease in EEG wave amplitude, a decrease in alpha band activity, and a gradual increase in theta or delta band activity. As the grading continues to increase, the EEG reveals an absence of brain wave activity (Oğün et al., 2002). Bariskaner et al. found that brain lactate and malondialdehyde concentrations positively correlate with EEG grading. Thus, when the brain lactate and malondialdehyde concentrations were suppressed after intravenous MgSO4 injection, the EEG grading decreased, EEG amplitude increased, alpha band activity increased, and delta band activity decreased (Bariskaner et al., 2003). As mentioned above, the decrease in alpha RP and increase in delta RP during the emergence period is one of the main EEG manifestations of ED in children. Therefore, we speculate that MgSO4 changes the delta/alpha ratio on the EEG during the emergence period by inhibiting the production of lactate and malondialdehyde in the brain, thus reducing ED occurrence. Further research is required to elucidate how lactate and malondialdehyde affect ion channels or protein receptors in neuronal cells, affecting EEG conduction and thus cortical EEG waveforms.

### 4.2 Increase in spindle waves

#### 4.2.1 Dexmedetomidine

Dexmedetomidine is a selective α2-adrenoceptor agonist with sedative, analgesic, anxiolytic, and sympatholytic effects. It may produce sedative effects by agonizing endogenous pro-sleep pathways through presynaptic mechanisms (Weerink et al., 2017). Under physiological conditions, the LC acts as an inhibitor on the hypothalamus-preoptic area (HPOA) via adrenergic neurons, inhibiting the brainstem and thalamus via GABAergic neurons. The activity of the brainstem reticular formation is associated with arousal. The LC also transmits excitation to the cerebral cortex via adrenergic neurons. The binding of dexmedetomidine to the α2 receptors hyperpolarizes LC neurons, decreasing norepinephrine release. This weakens HPOA inhibition by the LC, and neuron activation in the HPOA inhibits the brainstem arousal center, while cortical excitability decreases, resulting in a sedated state in patients (Brown et al., 2010; Brown et al., 2011). Recently, an increasing number of research related to dexmedetomidine and ED have demonstrated its diverse positive effects on ED (Yao et al., 2015; Lin et al., 2017; Sun et al., 2017; Sadeghi et al., 2022).

ENT surgery is a known risk factor for pediatric ED. In 2020, a meta-analysis by Jiao et al. showed that dexmedetomidine reduced the incidence of ED in children undergoing adenotonsillectomy under sevoflurane anesthesia and was superior to other drugs (Jiao et al., 2020). However, large, high-quality RCTs are required to confirm its superiority, because the included studies did not standardize the administration dose, route, or timing, and did not limit the age of inclusion. A subsequent RCT conducted by Lin et al. demonstrated that intravenous dexmedetomidine 1 μg/kg for maintenance of anesthesia in children undergoing odontotherapy significantly reduced the incidence of ED (Lin et al., 2017). Dexmedetomidine can be administered intranasally and intravenously. In an RCT conducted among 90 children aged 3–7 years who underwent unilateral strabismus surgery, Yao and colleagues found that intranasal administration of dexmedetomidine 1 μg/kg and 2 μg/kg 45 min before induction reduced the incidence of pediatric ED (Yao et al., 2015). Sadeghi et al. reported that, for ED prevention, intravenous dexmedetomidine 1 μg/kg 10 min before the end of adenotonsillectomy or cleft palate repair surgery was superior to the equivalent dose 10 min after the start of surgery (Sadeghi et al., 2022). Besides the administration route and timing, the dose is also a factor influencing the effect of dexmedetomidine. Intravenous administration of dexmedetomidine 0.25 μg/kg, 0.5 μg/kg, and 1.0 μg/kg 10 min before the end of laparoscopic hernia repair in children prevented ED occurrence—the fewest cases occurred in the 1.0 ug/kg group (Sun et al., 2017).

In 2016, Akeju et al. assessed EEG changes throughout the cortex during intravenous infusion of dexmedetomidine 1 μg/kg (within 10 min) followed by continuous infusion at 0.7 μg/kg/h for 50 min in eight healthy adult volunteers aged 18–36 years by 64-channel EEG. They found an increase in delta oscillations, a decrease in beta oscillations, an increase in occipital theta oscillations, and an increase in frontal spindle waves. These changes are similar to those in the second phase of NREM. They further found that intravenous dexmedetomidine 0.5 μg/kg or 1 μg/kg alone promoted NREM stage III in a dose-dependent manner (Akeju et al., 2018). The NREM stage III EEG waves were dominated by delta waves. Dexmedetomidine prolongs NREM and reduces wakefulness time, and this effect is dose-dependent (Feng et al., 2018). In 2020, Chamadia et al. found that oral dexmedetomidine increased the duration of NREM stage II and decreased the duration of REM sleep in an RCT conducted among 15 adults. They also suggested that dexmedetomidine promotion of NREM stage II or III may be related to the route and time of administration (Chamadia et al., 2020).

Overall, EEG characteristics after dexmedetomidine administration are unclear in children. Therefore, we hypothesize that dexmedetomidine may increase EEG spindle waves and prolong N2 sleep duration. Although delta oscillations may also increase, low CNS excitability persists, thereby preventing ED occurrence.

### 4.3 Decreased brain connectivity

#### 4.3.1 Ketamine

Ketamine is a non-competitive NMDA receptor antagonist with dose-dependent sedative and analgesic effects (Mihaljević et al., 2020). The analgesic action of ketamine is multimodal and mainly produced by blocking NMDA receptors in the spinal cord (Oye et al., 1992); secondly, it acts on opioid receptors to produce analgesic effects (Pacheco Dda et al., 2014). More studies on ketamine are emerging, aimed at preventing pediatric ED (Dalens et al., 2006; Ali et al., 2020).

Ali et al. analyzed the data of 120 children aged 3–15 years who underwent adenotonsillectomy and found that the incidence of ED in children who were given 0.15 mg/kg ketamine intravenous 10min before the end of the operation was 13.33% and that in the control group was 48.33% (Ali et al., 2020). Similarly, a randomized controlled study involving 90 children undergoing MRI found that 0.25 mg of ketamine at the end of surgery reduced the incidence of ED by about 13% compared with a control group (Dalens et al., 2006). However, a study by Schmitz et al., involving 351 children aged 3 months to 10 years undergoing MRI, found no difference in the incidence of ED between ketamine given 1 mg/kg at induction of anesthesia and the control group (Schmitz et al., 2018). In the former study, sevoflurane was used for induction and maintenance of anesthesia, while propofol was used for maintenance of anesthesia in the latter, and the incidence of ED after propofol was lower than that after sevoflurane. Secondly, different doses of ketamine, different time points of administration, and different ages of included children may cause inconsistent conclusions.

Lee et al. observed changes in frontal and parietal EEG among 30 surgery patients after intravenous induction with ketamine 2 mg/kg, and found an increase in delta, theta, and gamma RP (25–35 Hz) and a decrease in alpha and beta RP, with diminished brain connectivity (Lee et al., 2013). In 2015, by observing changes in the EEG of rats under ketamine anesthesia, Pal et al. found that gamma activity in the mid-high range (65–175 Hz) was significantly decreased after ketamine-induced loss of consciousness compared to the awake state; activity in other frequency bands did not markedly change. Delta activity was decreased and gamma wave activity was increased during the emergence period. They also found that cortical connectivity was reduced under ketamine anesthesia (Pal et al., 2015). An EEG after ketamine sedation is characterized by beta and gamma oscillations (Purdon et al., 2015). From the abovementioned study, we found that low-frequency gamma activity is a characteristic EEG manifestation of ketamine sedation. Although the EEG pattern under ketamine action is relatively active and different from that of propofol, Lee et al. suggested that both ketamine and propofol result in reduced frontoparietal connectivity and thus a sedated, unconscious state.

We hypothesize that cortical connectivity is reduced after intravenous ketamine administration, which reduces CNS excitability even though EEG activity appears relatively active. Ketamine may prevent ED occurrence by reducing the cortical connectivity of the child and its analgesic effect.

### 4.4 Pain

#### 4.4.1 Opioids

Opioids can be divided into three categories: natural (morphine, codeine, papaverine), synthetic (methadone, meperidine, fentanyl, alfentanil, sufentanil, remifentanil), and semisynthetic (hydromorphone). They produce analgesic effects by acting on μ, κ, and δ receptors. Opioids are widely used in clinical practice and are one of the essential drug classes used for general anesthesia. Recently, several opioids have been used to prevent ED in children (An et al., 2017; Kim et al., 2017; Choi et al., 2018; Chu and Pan, 2018).

Fentanyl is commonly used in pediatric anesthesia. In 2017, Kim et al. analyzed 10 RCTs and found that intravenous fentanyl before and after the end of surgery reduced the incidence of pediatric ED with sevoflurane general anesthesia (Kim et al., 2017). In addition to fentanyl, remifentanil, and sufentanil are commonly used in pediatric anesthesia and positively affect ED incidence. An RCT by Choi et al. revealed that, in children aged 3–7 years, remifentanil 0.05 μg/kg/min infusion at the end of strabismus surgery after discontinuation of sevoflurane, the incidence of ED was reduced by 35% (Choi et al., 2018). It has also been reported that administration of 0.1 mg/kg dezocine at the end of surgery has reduced the incidence of ED in children undergoing laparoscopic inguinal hernia repair (An et al., 2017) and that intravenous infusion of 0.01 mg/kg hydromorphone 10 min before surgery prevented ED in children undergoing strabismus surgery (Chu and Pan, 2018).

We believe that the mechanism of opioid prevention of ED is inextricably linked to its analgesic effect, which reduces the incidence of ED by suppressing pain, reduces sensitivity to external stimuli, and more closely resembles the natural arousal of NREM sleep.

#### 4.4.2 Acupuncture

Acupuncture is a type of acupuncture therapy that involves the insertion of fine needles into acupuncture points on the skin to a certain depth and for a sustained period of time in order to alleviate symptoms and cure diseases (Nakajima et al., 2022). In recent years, some studies have reported that acupuncture therapy can prevent the occurrence of postoperative ED in pediatric patients. Yuanchi Lin et al. observed 80 children aged 1–6 years who underwent tympanotomy with tympanic ventricular tubing, and acupuncture treatment was performed for 10 min at bilateral Hegu and Senmen points after induction of sevoflurane anesthesia, and it was found that the acupuncture therapy significantly reduced the severity of postoperative pain and agitation in children (Lin et al., 2009). However, contrastingly, a study by Martin et al. employed acupuncture at bilateral HT-7 and ear shen men points of children after anesthesia induction until the end of surgery and showed no significant difference in ED incidence between the two groups (Martin et al., 2020). The discrepancy in results may be due to the difference in acupuncture points and duration. Some studies have shown that acupuncture therapy may activate the release of opioid peptides in the adult CNS (Chernyak and Sessler, 2005; Zhao, 2008). Others have suggested that it can affect the release of visceral pain–modulating substances, such as increasing the secretion of β-endorphin and decreasing epinephrine, cortisol, and prostaglandin E2 levels (Lee et al., 2019). We believe that acupuncture therapy may prevent ED occurrence by reducing pain. However, it is unclear how it affects EEG changes, and the optimal acupuncture site, timing of initiation, and duration of treatment for the prevention of pediatric ED need to be further explored.

In summary, these interventions alter the EEG during wakefulness through their respective sedative and analgesic effects, increase the power of spindle or alpha waves, reduce the connectivity of t the brain, so that the EEG during the emergence period will be closer to the natural awakening EEG changes, and render the childless perceptive to external stimuli and reduce CNS excitability, thereby preventing the occurrence of pediatric ED. Furthermore, other interventions such as colistin, benzodiazepines, melatonin, gabapentin (Pinto Filho et al., 2019; Archana et al., 2022; Yang et al., 2022) and recorded maternal voice during emergence, and regional block can reduce the incidence of ED (Byun et al., 2018; Zhong et al., 2018; Yang et al., 2020). Interestingly, after intravenous administration of clonidine to achieve sedation in healthy volunteers, their EEG showed a decrease in dominant alpha activity and an increase in delta oscillations (Bischoff et al., 2004; Miyazaki et al., 2004). However, this EEG change is inconsistent with our previous findings that the increase in delta RP and decrease in alpha RP are associated with a high ED incidence. Nevertheless, sufficient clinical evidence supports the beneficial effect of clonidine on ED outcomes; more relevant studies are required to elaborate on the EEG changes after clonidine administration in children.

## 5 Conclusion

Based on the EEG level analysis, we concluded that the mechanism affecting ED occurrence is not unique but a combination of several factors. We hypothesize that the phenomena of increased EEG delta and alpha RP ratio, decreased spindle wave power, increased cerebral connectivity, increased neuronal excitability during anesthesia emergence and epileptiform discharges during anesthesia induction can be used as indicators for identifying ED, which allows us to target the administration of drugs related to the prevention of ED and to change the EEG manifestations of the affected children to prevent the occurrence of ED. In addition, the neurophysiological development of the child himself and factors such as pain are also associated with the development of ED. However, at present, our understanding of the bottom-up process from neural activity to behavior is very incomplete; and at the mechanism level, the causal relationship between EEG and ED performance has not yet been clearly established; the detailed mechanism of preventive medications and therapeutic EEGs in children, as well as the detailed physiological mechanisms behind the mechanism of EEGs in children with ED, have yet to be investigated, and more basic and clinical studies are needed in the future.
